# Multi‐Target Mechanisms of the Naofucong in Ameliorating Diabetes‐Associated Cognitive Dysfunction via cAMP/PKA/CREB‐Mediated Synaptic and Inflammatory Regulation

**DOI:** 10.1002/cns.70716

**Published:** 2025-12-23

**Authors:** Mei Ma, Yue Tian, Ruiying Yin, Guangchan Jing, Mengren Zhang

**Affiliations:** ^1^ Department of Traditional Chinese Medicine, Peking Union Medical College Hospital Peking Union Medical College and Chinese Academy of Medical Sciences Beijing China

**Keywords:** cAMP/PKA/CREB, DACD, multi‐omics, neuroinflammation, NFC, synaptic plasticity

## Abstract

**Objective:**

Diabetes‐associated cognitive dysfunction (DACD) is a prevalent and debilitating complication of diabetes, yet effective therapies remain limited. The traditional Chinese medicine compound Naofucong (NFC) has shown neuroprotective potential, but its underlying mechanisms are not fully understood. This study investigated the therapeutic efficacy and molecular mechanisms of NFC against DACD using an integrated multi‐omics approach combined with experimental validation.

**Methods:**

Streptozotocin‐induced DACD rats received NFC (22.5 g/kg/day) for 12 weeks. Cognitive performance was assessed by the Morris water maze. Transcriptomics and untargeted metabolomics were integrated to identify key regulatory pathways, which were further validated using immunofluorescence, Golgi staining, cytokine profiling, qPCR, and western blotting.

**Results:**

NFC significantly improved spatial learning and memory, attenuated neuronal damage in the hippocampus and cortex, and reduced pathological protein accumulation (APP, phosphorylated Tau). By integrating transcriptomics and metabolomics, we elucidated that NFC primarily acts via activation of the cAMP/PKA/CREB signaling pathway, leading to synaptic repair and neuroinflammatory modulation. Mechanistically, NFC restored synaptic ultrastructure, enhanced dendritic complexity and spine maturation, and upregulated neurotrophic factors (BDNF, NGF) and synaptic proteins (PSD‐95, SYN). Furthermore, NFC inhibited glial overactivation, decreased pro‐inflammatory cytokines (IL‐1β, TNF‐α, IFN‐γ, IL‐6, KC/GRO), and increased anti‐inflammatory cytokines (IL‐10, IL‐13, IL‐4), thereby re‐establishing neuroimmune balance.

**Conclusion:**

NFC exerts multi‐target neuroprotective effects by activating the cAMP/PKA/CREB pathway and coordinately regulating synaptic plasticity and neuroinflammation. These findings highlight NFC as a promising candidate for DACD treatment.

## Graphical abstract

Naofucong exerts multi‐target neuroprotective effects by activating the cAMP/PKA/CREB pathway and coordinately regulating synaptic plasticity and neuroinflammation.

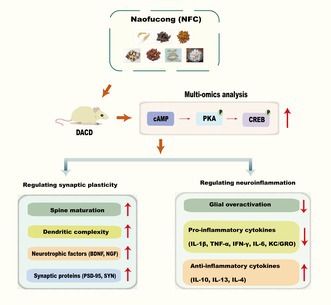

## 1 Introduction

Diabetes and its complications represent a major global health burden, with the prevalence of type 2 diabetes mellitus (T2DM) projected to exceed 1.3 billion cases by 2050 [1, 2]. Among its central nervous system complications, diabetes‐associated cognitive dysfunction (DACD) is increasingly recognized for its high prevalence, irreversibility, and strong link to dementia risk. Patients with T2DM have a 1.5–2‐fold higher likelihood of developing dementia and progress more rapidly from mild cognitive impairment to Alzheimer's disease compared with non‐diabetic individuals [3]. DACD is characterized by progressive deficits in learning and memory, underpinned by insulin resistance, tau hyperphosphorylation, impaired synaptic plasticity, and neuroinflammation [4, 5]. Current therapeutic strategies remain inadequate: intensive glycemic control provides only limited cognitive benefit, while monotherapies targeting amyloid‐β or tau have repeatedly failed in clinical trials [6, 7]. These challenges underscore the urgent need for multi‐target therapeutic approaches.

Traditional Chinese medicine (TCM) offers unique advantages in addressing complex chronic and neurodegenerative diseases through its multi‐component and multi‐target properties. Naofucong (NFC), a classical TCM formula comprising Panacis ginseng radix, Salviae miltiorrhizae radix, Polygoni multiflori radix Praeparata, Hirudo medicinalis, Poria, Coptidis rhizoma, and Acori tatarinowii rhizoma, has shown efficacy in ameliorating DACD in experimental models [8, 9, 10]. Previous studies suggest that NFC exerts neuroprotective effects by inhibiting apoptosis pathways, reducing oxidative stress and mitochondrial damage, and promoting amyloid‐β clearance [8, 9, 10]. However, the comprehensive pharmacological network underlying its action remains poorly defined, limiting its translational potential.

This study systematically investigates the neuroprotective mechanisms of NFC in DACD using an integrated transcriptomic and metabolomic strategy combined with experimental validation. Our findings highlight activation of the cAMP/PKA/CREB signaling pathway as a central mechanism through which NFC restores synaptic ultrastructure, enhances dendritic spine maturation, upregulates neurotrophic and synaptic proteins, and re‐establishes neuroimmune homeostasis. This study provides experimental evidence for a dual mechanism of “synaptic repair‐immune regulation,” supporting NFC as a promising multi‐target therapeutic candidate for DACD.

## 2 Materials and Methods

### 2.1 Preparation of NFC Decoction

The NFC is composed of seven Chinese medicinal materials: *Panacis ginseng radix*, *Salviae miltiorrhizae radix*, *Polygoni multiflori radix* Praeparata, *Hirudo medicinalis* , Poria, *Coptidis rhizoma*, *Acori tatarinowii rhizoma*, all procured from Beijing Tong Ren Tang Medicinal Materials Company (the specific dosage of each herb and the drug batch number are as follows: Table S1). All herbs were authenticated according to the Chinese Pharmacopeia (Beijing Tong Ren Tang Herbal Medicine Co. Ltd.). Herbs were decocted twice (40 and 20 min at 100°C), and the extracts were combined, concentrated to 200 mL, and stored at −80°C. The decoction was freshly prepared weekly using a TCM decoction machine (YJCXE20). Based on previous dose–response studies [8], a dose of 22.5 g/kg/day was selected for in vivo experiments.

### 2.2 Animals, Ethics, and Treatment

Male Sprague–Dawley rats (6 weeks, 180–210 g) were purchased from Beijing Huafukang Biotechnology (license no. SYXK [Beijing] 2020‐0026). Rats were housed under SPF conditions (22°C–25°C, 55% ± 15% humidity, 12/12 h light/dark cycle) with ad libitum food and water. All experiments were approved by the Animal Welfare and Ethics Committee of Beijing Huafukang (approval no. HFK‐AP‐20240812) and conducted in accordance with the ARRIVE guidelines.

**FIGURE 1:**
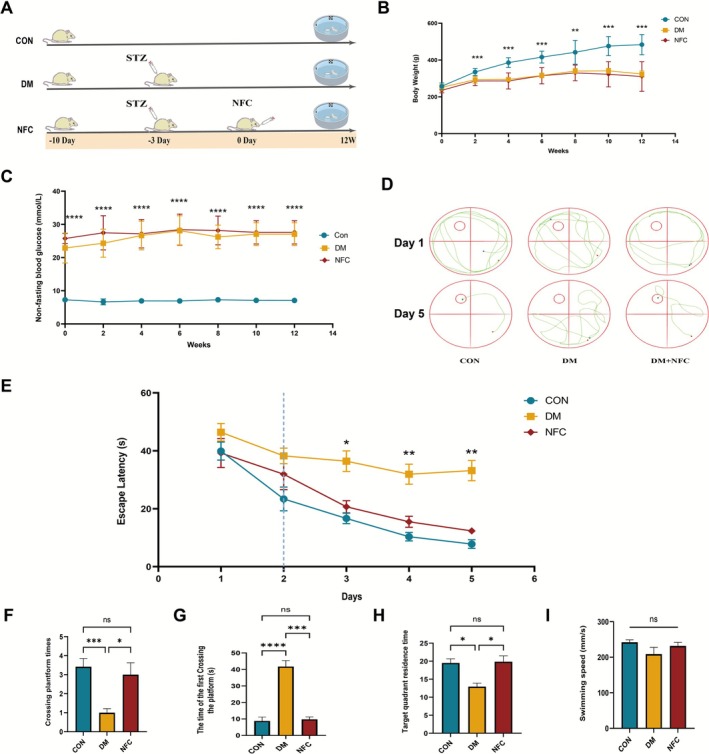
NFC ameliorates cognitive impairment in STZ‐induced diabetic rats. (A) Schematic illustration of the experimental design. (B) Body weight changes during the drug intervention period (*n* = 12). (C) Non‐fasting blood glucose levels during the drug intervention period (*n* = 12). (D) Representative swimming trajectories in the Morris water maze on Day 1 and Day 5 of the spatial probe test (*n* = 12). (E) Escape latency during the place navigation test (Days 1–5) (*n* = 12). (F) Number of platform crossings in the spatial probe test (*n* = 12). (G) Time to first platform crossing in the spatial probe test (*n* = 12). (H) Time spent in the target quadrant during the spatial probe test (*n* = 12). (I) Average swimming speed during the spatial probe test (*n* = 12). ns: not significant, *p < 0.05, **p < 0.01, ***p < 0.001, ****p < 0.0001.

After acclimatization, rats were randomly divided into three groups (*n* = 12): control (CON), diabetes model (DM), and NFC. Diabetes was induced by a single intraperitoneal injection of streptozotocin (STZ, 55 mg/kg, Solabio) in 0.45% solution; CON rats received citrate buffer (pH 4.5). Non‐fasting blood glucose ≥ 16.7 mmol/L at 72 h post‐injection was considered successful modeling. NFC rats were given NFC (22.5 g/kg/day, oral gavage) for 12 weeks (Figure 1A), while CON and DM groups received distilled water (1 mL/100 g/day). Body weight and blood glucose were measured biweekly.

### 2.3 Behavioral Testing

Spatial learning and memory were assessed using the Morris water maze (MWM) after 12 weeks of treatment. The apparatus consisted of a circular tank (180 cm diameter) with a hidden platform submerged 1 cm below the surface. From days 1–5, rats underwent four navigation trials per day. On day 6, the platform was removed for a probe test. Escape latency, platform crossings, time spent in the target quadrant, and swimming speed were recorded using the VisuTrack system. The experimenter was blinded to group allocation.

### 2.4 Tissue Collection

After behavioral testing, rats were fasted for 12 h and anesthetized with isoflurane. Blood was collected from the abdominal aorta, and serum was separated by centrifugation (3000 rpm, 20 min). Hippocampal and cortical tissues were dissected on ice, snap‐frozen in liquid nitrogen, and stored at −80°C. For histology, whole brains from three rats per group were fixed in 4% paraformaldehyde.

### 2.5 Histology and Immunofluorescence

Fixed brains were paraffin‐embedded and sectioned (5 μm). Sections were stained with hematoxylin–eosin (HE) and Nissl to evaluate neuronal morphology. For immunofluorescence, sections were incubated with primary antibodies (Table S2) overnight at 4°C, followed by fluorescent secondary antibodies and DAPI counterstaining. Images were acquired using a 3D HISTECH scanner (Pannoramic 250 FLASH), and quantification was performed with ImageJ by blinded investigators.

### 2.6 Transcriptomic Sequencing of Hippocampus

Total RNA was extracted from hippocampi (*n* = 6 per group). Libraries were prepared following a standard protocol and sequenced on an Illumina NovaSeq 6000 (PE150). Clean reads were aligned to the reference genome using HISAT2, and differential expression was analyzed with DESeq2 (|log_2_FC| > 2, *p* < 0.05). Gene Ontology (GO) and KEGG pathway enrichment analyses were performed.

### 2.7 Untargeted Metabolomics of Hippocampus

Untargeted metabolomics was performed on hippocampal tissue (*n* = 6 per group) using UPLC‐Q‐Exactive Orbitrap‐MS (Thermo Fisher Scientific) in positive and negative ESI modes. The conditions set were provided in the Supporting Information. Data were processed in MS‐DIAL, and metabolites were identified against HMDB, MassBank, and an in‐house database. Differential metabolites were defined as VIP > 1.5 and *p* < 0.05 and subjected to KEGG enrichment analysis.

### 2.8 Golgi Staining

Golgi‐Cox staining (Servicebio kit) was used to visualize dendritic morphology. Coronal sections (60 μm) were analyzed using ImageJ (NeuronJ and Sholl plugins). Spine density and morphology (mushroom, stubby, thin) were classified according to standard criteria [11, 12].

### 2.9 Transmission Electron Microscopy (TEM)

Hippocampal blocks (1 mm^3^) were fixed in glutaraldehyde, post‐fixed with osmium tetroxide, dehydrated, and embedded in resin. Ultrathin sections (60 nm) were stained with uranyl acetate and lead citrate and examined under a Hitachi HT7800 TEM at 40,000×. Random fields were imaged and analyzed by blinded investigators.

### 2.10 qPCR

Total RNA was extracted from hippocampi with TRIzol (Vazyme) and reverse‐transcribed into cDNA. qPCR was performed on a QuantStudio 5 system using SYBR Green Master Mix (Vazyme). Relative expression was calculated using the 2^−ΔΔCT^ method. Primer sequences are listed in Table S3.

### 2.11 Western Blotting

Hippocampal proteins were extracted with RIPA buffer and quantified with the BCA assay. Equal amounts were resolved by SDS‐PAGE, transferred to PVDF membranes, and probed with primary antibodies (Table S4). Signals were visualized using ECL (Yeasen) and quantified with ImageJ. GAPDH served as the loading control.

### 2.12 Statistical Analysis

Data are expressed as mean ± SEM. Statistical analyses were performed with GraphPad Prism 9. For two‐group comparisons, unpaired two‐tailed *t*‐tests or Mann–Whitney *U* tests were applied. For multiple groups, one‐way or two‐way ANOVA followed by Tukey's post hoc test was used. A *p* < 0.05 was considered statistically significant.

### 2.13 NFC Ameliorates Cognitive Impairment in STZ‐Induced Diabetic Rats

The experimental timeline is shown in Figure 1A. Body weight and non‐fasting blood glucose were monitored throughout the study. From week 2, DM and NFC rats exhibited significantly lower body weight than controls (Figure 1B), indicating that NFC did not affect body weight in diabetic rats. Non‐fasting blood glucose remained elevated in DM and NFC groups (20–30 mmol/L) and was significantly higher than CON (Figure 1C), confirming successful diabetes induction and NFC did not directly influence glucose levels.

In the MWM spatial navigation test, escape latency decreased over time in all groups. No significant differences were observed during the first two days; however, DM rats displayed longer escape latencies from day 3 onward compared to CON, reflecting impaired learning. NFC treatment reduced escape latency compared with DM rats on days 3–5 (Figure 1D,E). These results suggest that NFC treatment improves learning and memory function.

In the MWM probe test, DM rats exhibited fewer platform crossings, longer latency to first crossing, and reduced time in the target quadrant, suggesting impaired spatial memory. NFC treatment significantly increased platform crossings, reduced latency, and increased time in the target quadrant without affecting swimming speed, indicating that cognitive improvement was independent of motor function (Figure 1F–I).

### 2.14 NFC Alleviates Neuronal Damage and Pathological Protein Accumulation in Diabetic Rat Brains

**FIGURE 2:**
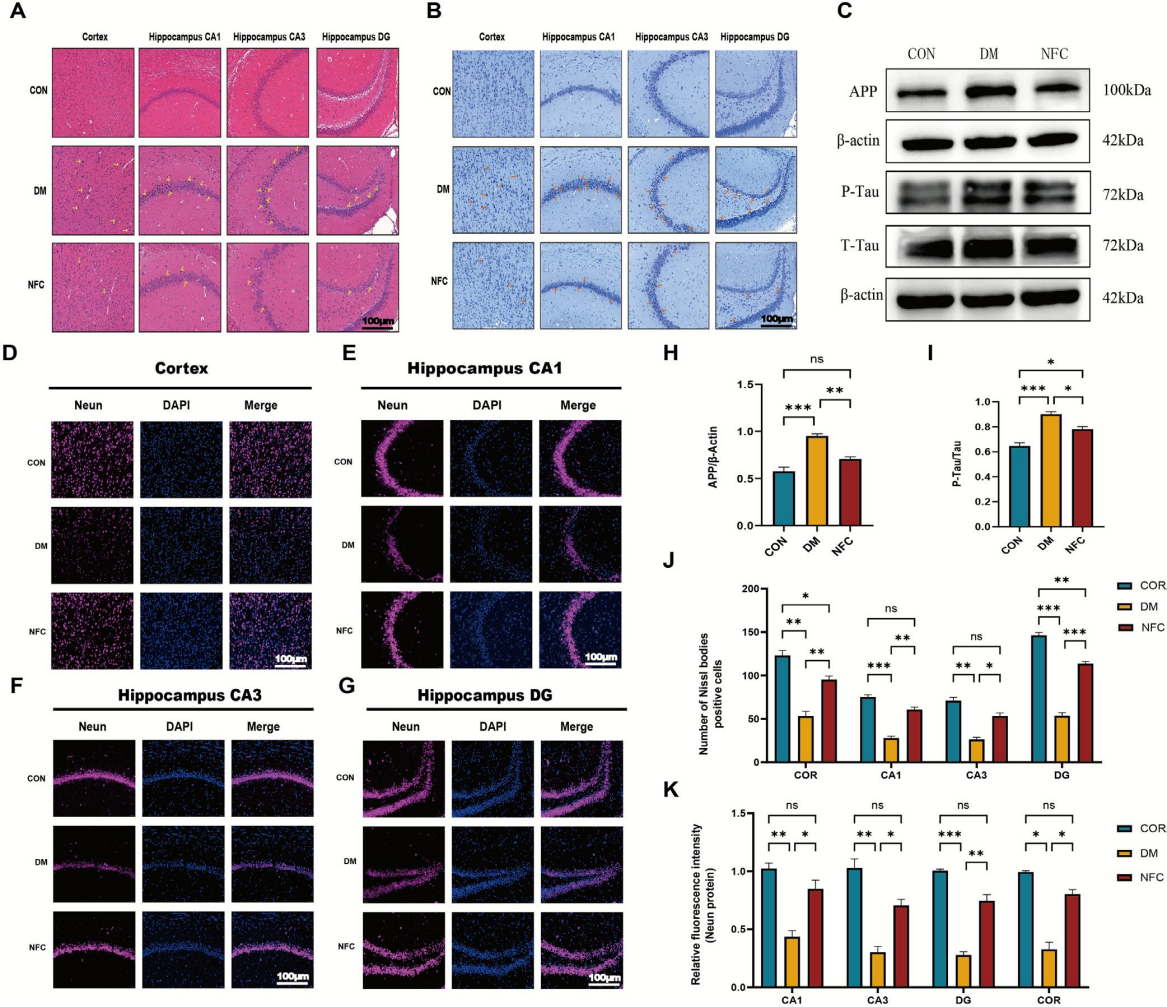
NFC alleviates neuronal damage and pathological protein accumulation in the brain of diabetic rats. (A) H&E staining of the cortex, hippocampal CA1, CA3, and DG regions (*n* = 3). Scale bar: 100 μm. (B, J) Nissl staining of the cortex, hippocampal CA1, CA3, and DG regions (*n* = 3). Scale bar: 100 μm. (C, H, I) Western blot analysis of APP and Tau (P‐Tau) in the hippocampus (*n* = 3). (D–G, K) Immunofluorescence staining for the neuronal marker NeuN in the cortex (D), hippocampal CA1 (E), CA3 (F), and DG (G) regions (*n* = 3). Scale bar: 100 μm. ns: not significant, *p < 0.05, **p < 0.01, ***p < 0.001, ****p < 0.0001.

Histological analysis of the hippocampus and cortex revealed that DM rats exhibited loosely arranged neurons, nuclear condensation, and pyramidal cell damage, while CON rats showed intact neuronal architecture (Figure 2A). NFC treatment improved neuronal arrangement and reduced nuclear condensation.

Nissl staining confirmed that DM rats had fewer, lightly stained Nissl bodies, whereas NFC significantly increased Nissl body number (Figure 2B,J). Immunofluorescence of NeuN showed reduced neuronal density in DM rats, which was restored by NFC (Figure 2D–G,K).

Western blot analysis indicated that DM rats had elevated hippocampal APP and P‐Tau levels, both of which were significantly reduced by NFC (Figure 2C,H,I). These results suggest that NFC mitigates diabetes‐induced neuronal loss and pathological protein accumulation.

### 2.15 Transcriptomic Alterations Induced by NFC in Diabetic Rats

**FIGURE 3:**
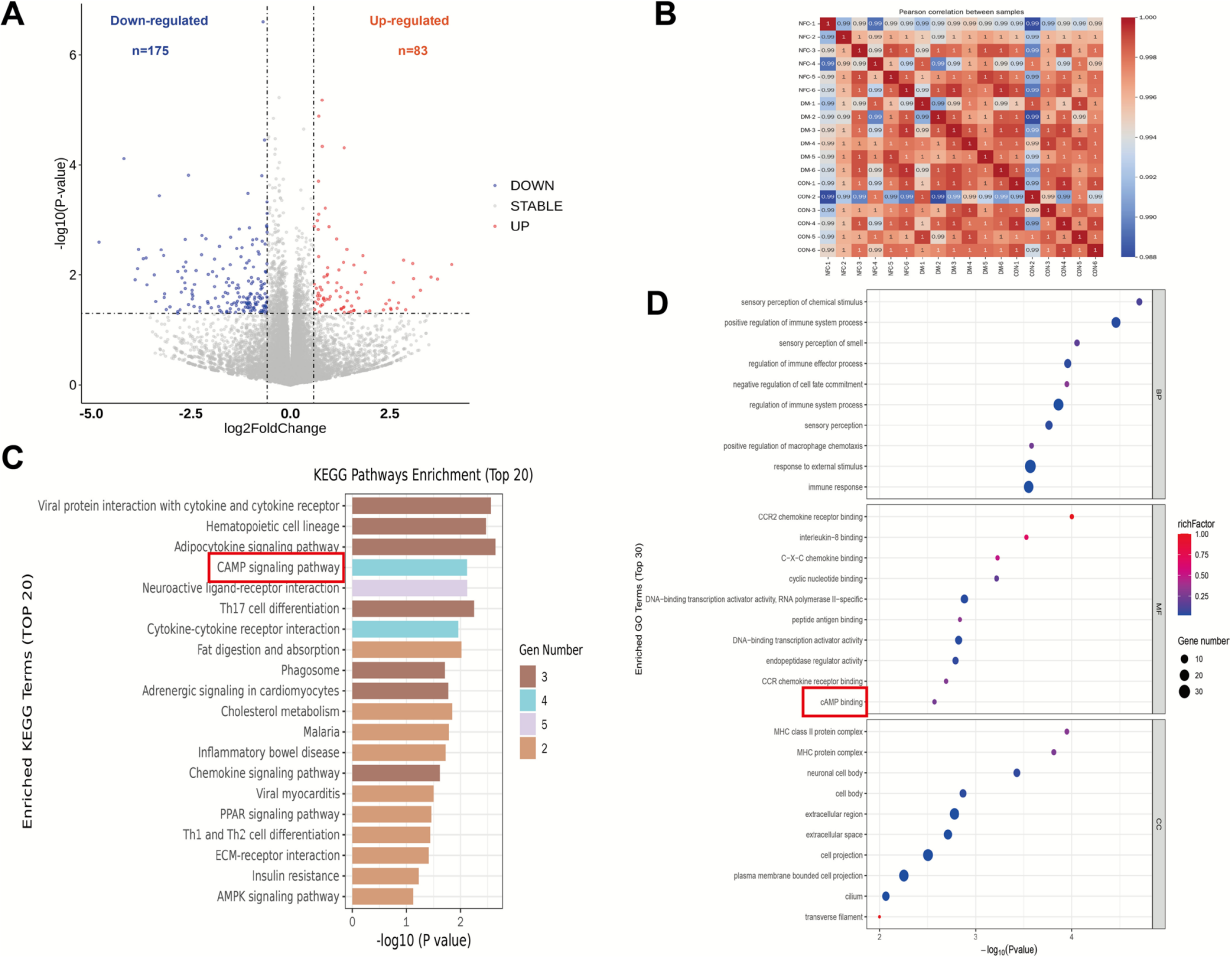
NFC treatment changed the transcriptomics of hippocampus in rats with DACD. (A) Volcano plot of differentially expressed genes (DEGs) in the hippocampus of DM versus NFC rats (*n* = 6). (B) Heatmap of correlation analysis between hippocampal samples. (C) Top 20 enriched KEGG pathways in DEGs. (D) The bubble plot of top 30 GO signaling pathways enriched by DEGs.

To further explore the mechanism by which NFC prevents DACD, we conducted transcriptomic analysis of hippocampal tissue from the DM and NFC groups. Transcriptomic profiling of hippocampal tissue revealed 258 differentially expressed genes (DEGs) following NFC treatment, including 83 upregulated and 175 downregulated genes (|log_2_FC| > 2, *p* < 0.05, Figure 3A). Pearson correlation analysis confirmed high inter‐sample consistency (Figure 3B), supporting the validity of downstream analysis. KEGG enrichment highlighted the cAMP signaling pathway, cytokine‐cytokine receptor interaction, chemokine signaling, neuroactive ligand‐receptor interaction, insulin resistance, and AMPK signaling (Figure 3C). GO analysis indicated enrichment in cAMP binding, neuronal cell body, cell projection, and inflammatory processes (Figure 3D). Cross‐analysis identified cAMP signaling and immune response as key pathways modulated by NFC.

### 2.16 Metabolomic Alterations Induced by NFC in Diabetic Rats

**FIGURE 4:**
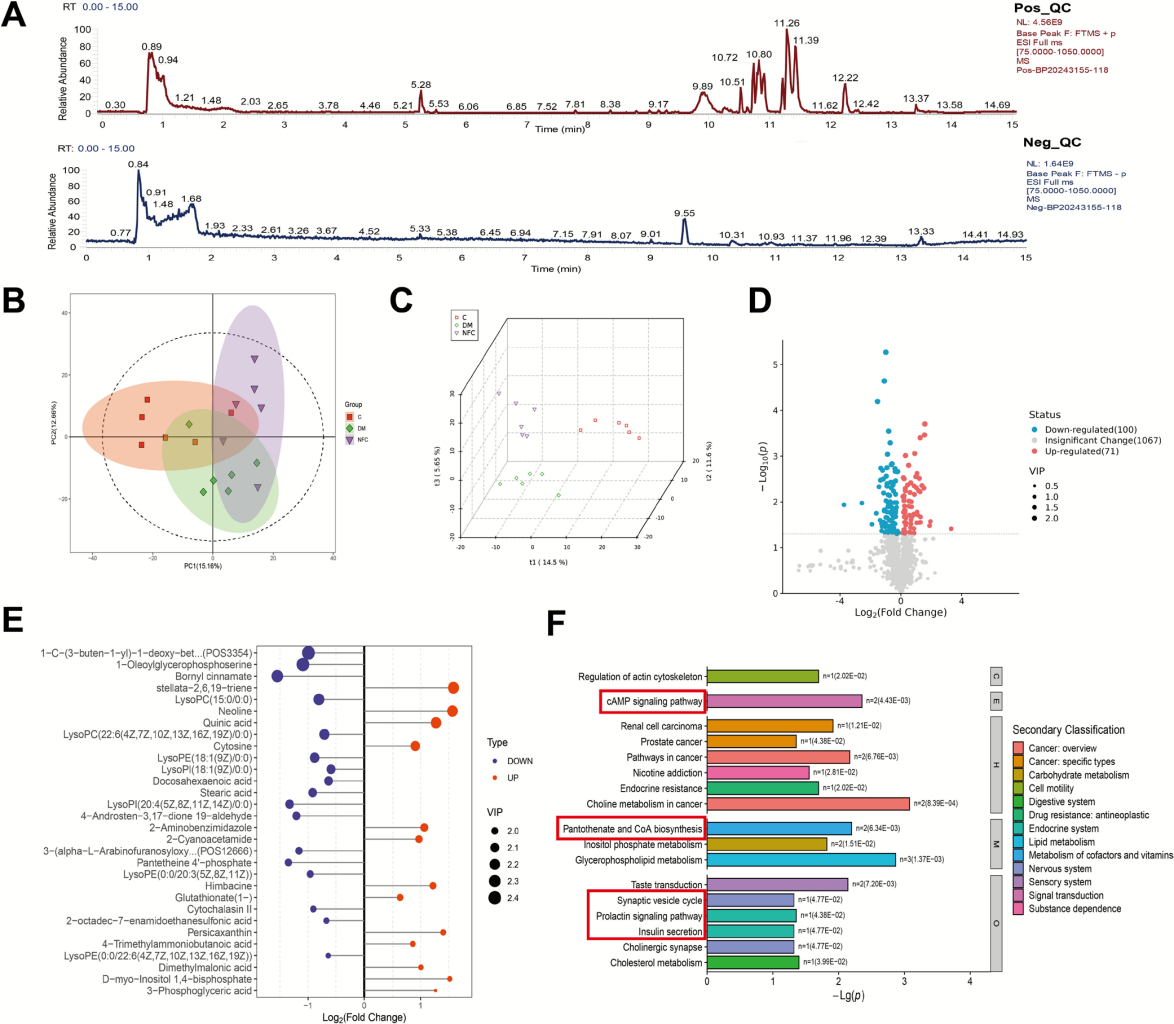
NFC treatment changed the Non‐targeted metabonomics of hippocampus in rats with DACD. (A) Representative BPI chromatogram of positive (Pos_QC) and negative (Neg_QC) quality control samples. (B) The PCA score plot. (C) The PLS‐DA 3D plot. (D) The volcanic map of differential metabolites. (E) The VIP top 30 difference multiple and VIP diagram. (F) The bar plot of KEGG pathway classification.

We conducted untargeted metabolomics analysis on hippocampal tissue from three rat groups to examine the regulatory effects of NFC on the metabolic alterations associated with DACD. Quality control (QC) samples exhibited consistent peak intensities and retention times in both positive and negative ion modes (Base Peak Chromatogram, Figure 4A), indicating minimal instrument variability and high data reliability. Principal component analysis (PCA) and partial least squares discriminant analysis (PLS‐DA) (Figure 4B,C) revealed clear separation between the DM and NFC groups, with robust model stability and strong discriminative power, supporting their suitability for differential metabolite screening. Untargeted metabolomics of hippocampal tissue identified 171 differentially expressed metabolites (71 upregulated, 100 downregulated, FC ≥ 1.5, *p* < 0.05, Figure 4D). Top metabolites included 1‐oleoylglycerophosphoserine, LPC (22:6), LPC (15:0), stellata‐2,6,19‐triene, and bornyl cinnamate, linked to neurotransmitter release, neuroinflammation, and antioxidant responses. KEGG analysis revealed modulation of the cAMP signaling pathway, cholinergic synapse, synaptic vesicle cycle, endocrine resistance, and insulin secretion (Figure 4E,F). These data suggest that NFC improves metabolic dysregulation associated with DACD.

### 2.17 Multi‐Omics Integration Indicates NFC Activates the cAMP/PKA/CREB Pathway

**FIGURE 5:**
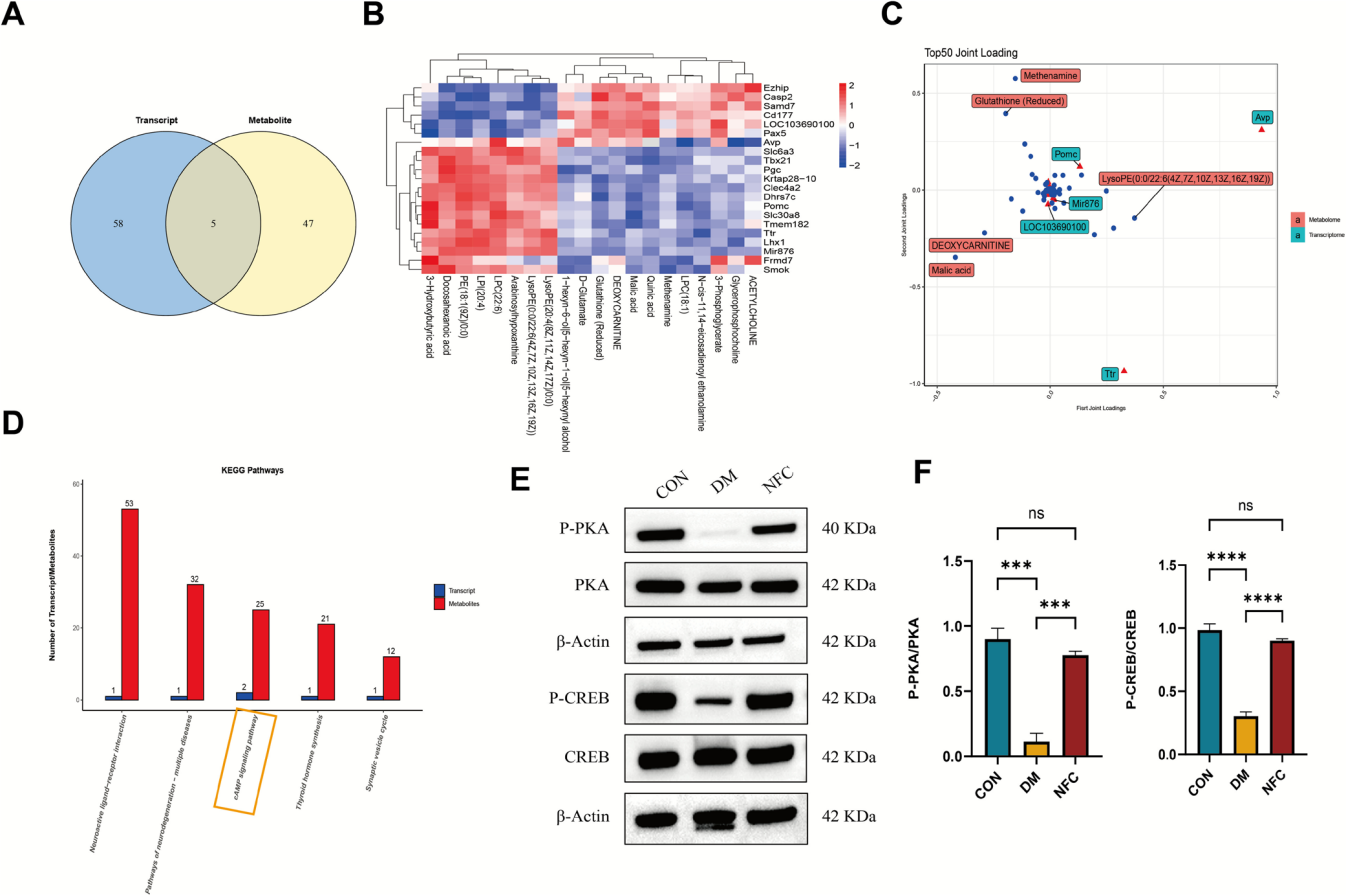
Multi‐omics integration analysis screened for key regulatory pathways involved in NFC‐mediated improvement of DACD. (A) Venn diagram of differential genes and metabolites. (B) Gene‐metabolite correlation heatmap. (C) The joint loadings diagram. (D) KEGG enrichment pathway diagram of differential genes and differential metabolism. (E) Schematic diagram of protein expression of PKA, p‐PKA, CREB, and p‐CREB in the hippocampal tissue of rats from all groups. (F) Western blot analysis of p‐PKA/PKA and p‐CREB/CREB in the hippocampal tissue of rats from all groups (*n* = 3). ns: not significant, ***p < 0.001, ****p < 0.0001.

Integration of DEGs and differential metabolites revealed five overlapping features, suggesting coordinated transcriptional and metabolic responses (Figure 5A,B). KEGG pathway enrichment showed that neuroactive ligand‐receptor interaction, neurodegeneration, cAMP signaling, thyroid hormone synthesis, and synaptic vesicle cycle were enriched (Figure 5C,D).

Notably, the cAMP signaling pathway was consistently enriched across transcriptomic, metabolomic, and integrated analyses. Western blot validation demonstrated that NFC restored phosphorylated PKA (P‐PKA) and CREB (P‐CREB) levels in DM rats (Figure 5E,F), confirming pathway activation as a key mechanism of cognitive protection.

### 2.18 NFC Enhances Synaptic Plasticity via cAMP/PKA/CREB Activation

To further validate NFC's role in enhancing synaptic plasticity via cAMP/PKA/CREB activation, we conducted a systematic analysis at ultrastructural, neuronal, and molecular levels.

**FIGURE 6:**
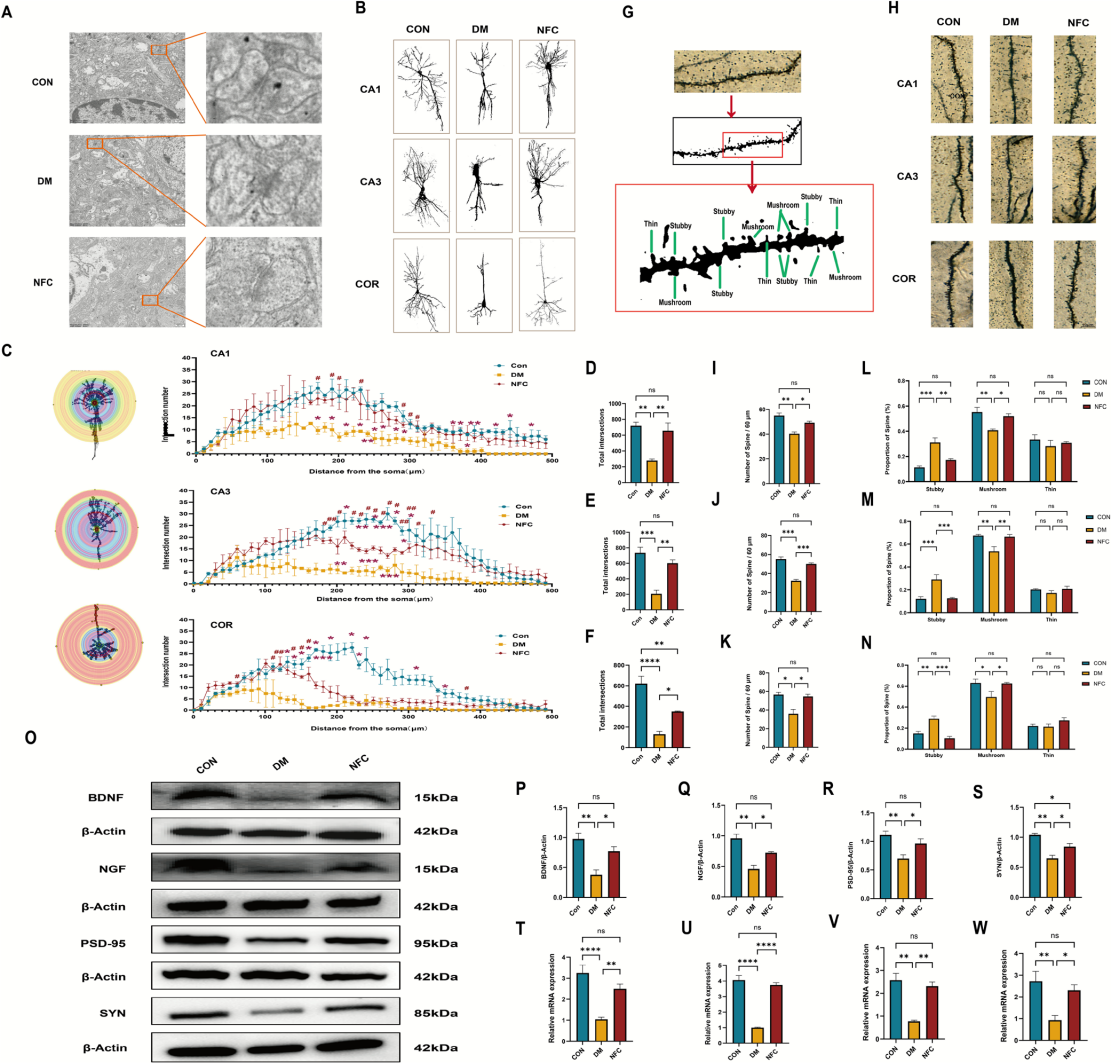
NFC enhances synaptic plasticity of DACD. (A) Ultrastructural observation of synapses in rats. Scale bar = 500 nm. (B) Cortex, hippocampal CA1 and CA3 neuronal images demonstrate dendritic branching. (C) Sholl plots: Dendritic arbor complexity in cortical, hippocampal CA1/CA3/DG neurons (across groups). *x*‐axis: distance from cell body; *y*‐axis: tracing intersections with Sholl shells. Insets: Representative neuron tracings overlaid on 10‐μm interval Sholl shell circles. DM vs. NFC, ^#^ *p* < 0.05, ^##^ *p* < 0.01, (D–F) Total neuronal intersections in cortex (D), hippocampal CA1 (E), and CA3 (F) (per group quantification). (G) Images showcasing labeled spines for examination. Spines including mushroom, thin, and stubby varieties are recognized according to structural criteria. (H) Exemplary dendritic segments: cortical, hippocampal CA1/CA3 neurons (per group, Scale bar = 10 μm). (I–K) Assessment of the density of dendritic spines in pyramidal neurons situated in the cortex (I), CA1 (J), and CA3 (K) regions. (L–N) Morphological categorization of dendritic spines in cortical, hippocampal CA1 (M), and CA3 (N) pyramidal neurons. (O–S) Schematic diagram and quantitative analysis of protein expression of BDNF, NGF, PSD‐95, and SYN in the hippocampal tissue of rats from all groups (*n* = 3). (T–W) MRNA expression of BDNF, NGF, PSD‐95, and SYN in the hippocampus of rats from all groups (*n* = 3). ns: not significant, *p < 0.05, **p < 0.01, ***p < 0.001, ****p < 0.0001.

Transmission electron microscopy revealed synaptic ultrastructural damage in DM rats, including disrupted membranes, swollen mitochondria, widened synaptic clefts, and reduced synaptic vesicles. NFC partially restored synaptic ultrastructure (Figure 6A).

Golgi staining showed that DM rats had reduced dendritic complexity and spine density, particularly mushroom spines. NFC treatment increased dendritic intersections, total spine density, and mushroom spine proportion, while reducing stubby spines (Figure 6B–N).

qPCR and Western blotting indicated that NFC restored hippocampal BDNF, NGF, PSD‐95, and SYN expression at both mRNA and protein levels (*p* < 0.05, Figure 6O–W), enhancing synaptic plasticity.

### 2.19 NFC Modulates Neuroinflammation to Improve DACD

Based on multi‐omics results, NFC may alleviate DACD by modulating neuroinflammation. Therefore, this study further explored its mechanisms through glial cell activation and inflammatory cytokine balance.

**FIGURE 7:**
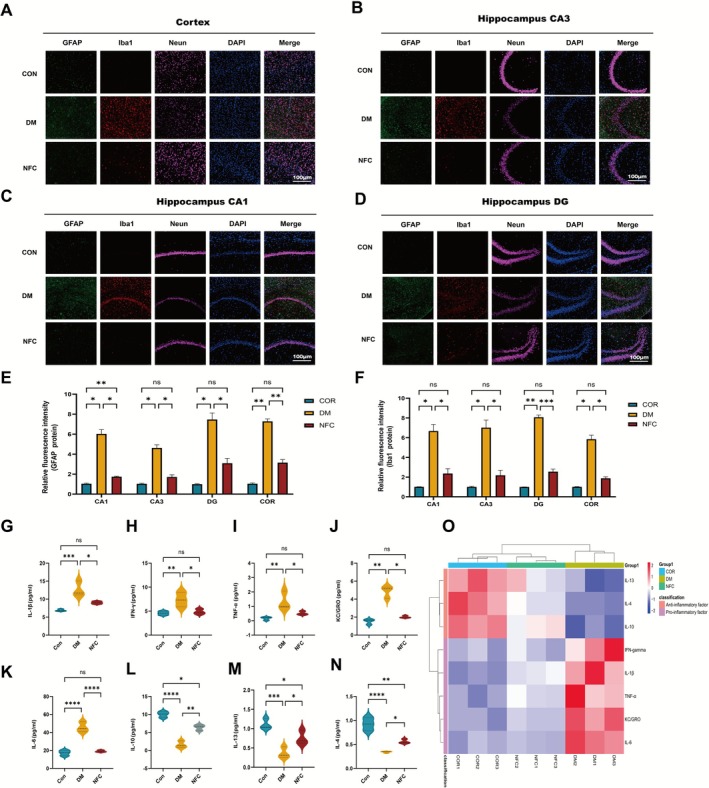
NFC regulates neuroinflammatory microenvironment. (A–D) Representative images of immunofluorescence of GFAP (green), IBA (red) and NenN (pink) in the cerebral cortex, hippocampal CA1, CA3, and DG regions of rats from all groups. DAPI (blue) indicates cell nuclei. (E, F) Fluorescence intensity of GFAP and IBA1 in the cerebral cortex, hippocampal CA1, CA3, and DG regions of rats from all groups (*n* = 3). (G–K) Pro‐inflammatory cytokines (IL‐1β, IFN‐γ, TNF‐α, IL‐6, KC/GRO) levels in the hippocampus of rats from all groups (*n* = 3). (L–N) Anti‐inflammatory cytokines (IL‐13, IL‐10, IL‐4) levels in the hippocampus of rats from all groups (*n* = 3). (O) Heatmap of cytokine expression in the hippocampus of rats from all groups (*n* = 3). ns: not significant, *p < 0.05, **p < 0.01, ***p < 0.001, ****p < 0.0001.

Immunofluorescence analysis showed that DM rats had elevated GFAP and Iba1 expression, with hypertrophic astrocytes and amoeboid microglia, indicating glial overactivation. NFC reduced glial activation and restored morphology (*p* < 0.05, Figure 7A–F).

Multiplex cytokine assays demonstrated that NFC decreased pro‐inflammatory cytokines (IL‐1β, TNF‐α, IL‐6, IFN‐γ, KC/GRO) and increased anti‐inflammatory cytokines (IL‐4, IL‐10, IL‐13) compared to DM rats (*p* < 0.05, Figure 7G–O), confirming its dual immunomodulatory effects.

## 3 Discussion

DACD is a central nervous system complication of diabetes, characterized by complex pathological mechanisms and limited therapeutic options. In this study, integrated multi‐omics analysis was employed to systematically investigate the neuroprotective effects and underlying mechanisms of NFC in DACD. For the first time, we demonstrated that NFC restores synaptic integrity and immune homeostasis through activation of the cAMP/PKA/CREB signaling pathway, providing novel insights into neuroprotective strategies for DACD.

The hippocampus, particularly the CA1, CA3, and DG subregions, is essential for learning and memory, orchestrating information encoding, consolidation, and retrieval [13, 14, 15]. Neuronal loss and pathological protein accumulation, such as APP and P‐Tau, are early hallmarks of cognitive impairment [16, 17]. In diabetic rats, hyperglycemia induced structural damage in the hippocampus and cortex, resulting in neuronal loss. NFC treatment attenuated these morphological abnormalities and significantly reduced APP and P‐Tau expression, suggesting early molecular protection.

Synapses are the primary sites for neuronal communication and memory encoding [18, 19, 20]. In DACD, hyperglycemia disrupts synaptic ultrastructure, reduces dendritic complexity, and impairs dendritic spine density, particularly mushroom spines, which are critical for stable synaptic connections. NFC intervention repaired synaptic ultrastructure, increased dendritic branching, and restored spine density and morphology. These effects support enhanced synaptic plasticity and cognitive function. Dendritic spine remodeling, from stubby to mushroom spines, is closely linked to synaptic activity and neurotrophic factor expression [21, 22]. NFC promoted this maturation, reinforcing its role in restoring functional connectivity.

The cAMP/PKA/CREB signaling pathway regulates dendritic spine dynamics, synaptic protein expression, and neurotrophic factor production [23, 24, 25, 26, 27]. PSD‐95, as a structural protein of the postsynaptic density, stabilizes the postsynaptic membrane and modulates receptor phosphorylation, thereby enhancing synaptic transmission [28]. It is a key marker of synaptic maturation; reduced expression can result in disrupted synaptic architecture and impaired signaling [28]. Additionally, SYN is located on the presynaptic membrane, where it regulates vesicle mobilization and neurotransmitter release efficiency by binding to synaptic vesicles and the cytoskeleton. After being phosphorylated by PKA, it can release vesicles from their constraints, increasing neurotransmitter release and enhancing synaptic transmission [29, 30]. Multi‐omics analyses in this study identified significant enrichment of the cAMP pathway, which was further validated by Western blotting showing restoration of P‐PKA and P‐CREB in NFC‐treated rats. Upregulation of BDNF, NGF, PSD‐95, and SYN at both mRNA and protein levels confirms that NFC enhances synaptic plasticity via this pathway.

Neuroinflammation is another key contributor to DACD linking metabolic dysregulation to synaptic and neuronal damage [31]. Emerging research suggests that microglia, innate immune cells of the central nervous system, and astrocytes are crucial for the synaptic pruning process [32, 33]. In this study, glial overactivation was evidenced by increased GFAP^+^ astrocytes and Iba1^+^ microglia, accompanied by elevated pro‐inflammatory cytokines (IL‐1β, TNF‐α, IL‐6, IFN‐γ, KC/GRO) and reduced anti‐inflammatory cytokines (IL‐4, IL‐10, IL‐13) in diabetic rats. This observation aligns with previous reports indicating that diabetes promotes excessive synaptic phagocytosis by microglia and impairs astrocyte function [34, 35, 36]. From the perspective of the “synapse‐immune” cross‐talk, under physiological conditions, microglia and astrocytes maintain synaptic‐immune homeostasis by precisely pruning abnormal synapses and providing trophic support, thereby preserving cognitive function. In pathological states such as diabetes, this balance is disrupted. Excessive immune activation and synaptic injury form a vicious cycle that drives the progression of cognitive impairment. Overactivated microglia release pro‐inflammatory factors (such as TNF‐α, IL‐1β, etc.), which can disrupt synaptic homeostasis through multiple mechanisms: on the one hand, they can directly damage the structure of the postsynaptic dense zone, inhibit BDNF expression, and thereby block dendritic spine maturation, which is highly consistent with the results of reduced mushroom spines in diabetic model rats in this study [37]. In addition, these cytokines suppress neurogenesis in the hippocampal dentate gyrus, induce mature neuronal apoptosis, and impair connectivity in key cognitive circuits such as the hippocampus‐prefrontal cortex, leading to memory encoding deficits and impaired learning [38, 39]. Therefore, targeting key links in the “synapse‐immune” cross‐talk (such as inhibiting excessive activation of glial cells and regulating the balance of inflammatory factors) is an important strategy for improving DACD. This mechanism underscores the unique advantage of NFC in restoring immune‐synapse balance through multi‐target modulation, thereby exerting neuroprotective effects.

Importantly, NFC's neuroprotective effects appear independent of glucose regulation. While hyperglycemia initiates DACD, non‐glucose‐dependent mechanisms, including neuroinflammation, synaptic plasticity impairment, and glial dysfunction play central roles in cognitive decline [5, 40, 41, 42]. NFC directly modulates these pathological processes, offering therapeutic potential even in patients with well‐controlled blood glucose but ongoing cognitive impairment.

This study has several limitations. First, although multi‐omics analyses identified the cAMP pathway as a key target and its activation was validated by Western blotting, pathway‐specific inhibition using cAMP antagonists (e.g., H‐89 or Rp‐cAMPS) was not performed. Second, in vitro validation of NFC's direct effects on neuronal phenotypes, including synaptic plasticity and neuroinflammation, was not conducted. Future studies should address these limitations to strengthen mechanistic conclusions.

## 4 Conclusion

**FIGURE 8:**
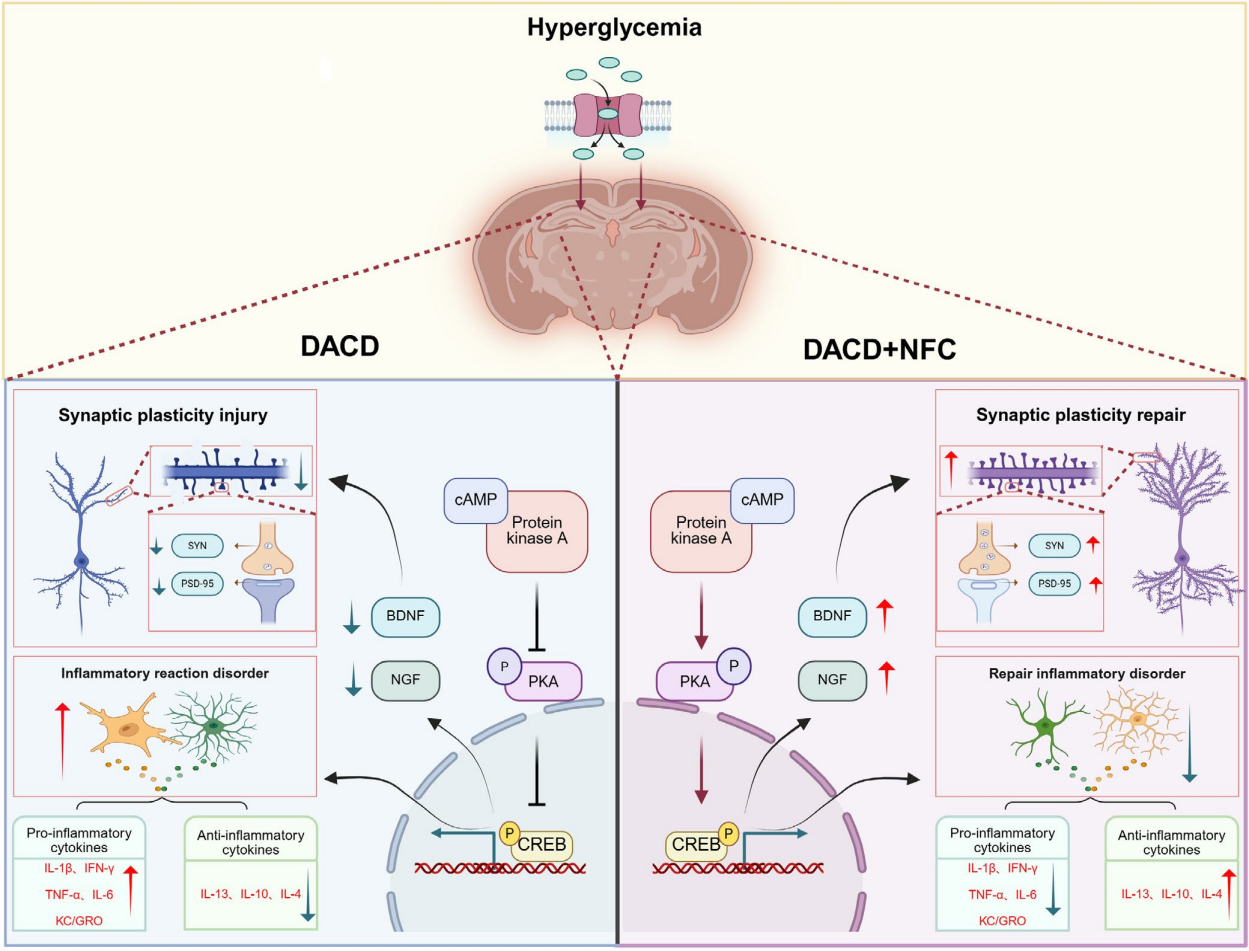
NFC activates the cAMP/PKA/CREB signaling pathway to synergistically regulate the dual axis of synaptic repair and immune homeostasis.

In summary, the role of NFC in improving cognitive impairment in a rat model of DACD by activating the cAMP/PKA/CREB signaling pathway, thereby coordinating synaptic repair and immune homeostasis (Figure 8). At the synaptic level, NFC restores ultrastructural integrity, enhances dendritic complexity, increases mature mushroom spine density, and upregulates synaptic proteins (PSD‐95, SYN) and neurotrophic factors (BDNF, NGF), collectively improving synaptic plasticity. At the immune level, NFC suppresses aberrant activation of glial cells (GFAP^+^ astrocytes, Iba1^+^ microglia) and restores neuroinflammatory balance by downregulating pro‐inflammatory cytokines (IL‐1β, TNF‐α, IL‐6) and upregulating anti‐inflammatory cytokines (IL‐10, IL‐4). These findings suggest that NFC is a potential multi‐target therapeutic candidate for DACD and provide experimental evidence supporting its application in the prevention and treatment of DACD.

## Author Contributions

**Mei Ma:** writing – original draft preparation, writing – reviewing and editing. **Yue Tian:** writing – original draft preparation, writing – reviewing and editing. **Ruiying Yin:** editing. **Mengren Zhang:** conceptualization, supervision. **Guangchan Jing:** conceptualization, supervision. All authors have read and approved the final manuscript.

## Funding

The authors have nothing to report.

## Ethics Statement

Male Sprague–Dawley rats (6 weeks old, 180–210 g) were obtained from Beijing Huafukang Biotechnology Co. Ltd. (license number: SYXK [Beijing] 2020‐0026). All procedures were approved by the Animal Welfare and Ethics Committee of Beijing Huafukang Biotechnology Co. Ltd. (approval number: HFK‐AP‐20240812).

## Consent

All authors have given their final approval for the manuscript to be published and have no objections to the publication of any details presented in the manuscript, such as images, videos, and recordings.

## Conflicts of Interest

The authors declare no conflicts of interest.

## Supporting information


**Table S1:** cns70716‐sup‐0001‐AppendixS1.docx.

## Data Availability

Data will be made available on request.
