# Supplementary material for: Multi‐Target Mechanisms of the Naofucong in Ameliorating Diabetes‐Associated Cognitive Dysfunction via cAMP/PKA/CREB‐Mediated Synaptic and Inflammatory Regulation
Source: CNS Neurosci Ther. 2025 Dec 23;31(12):e70716. doi: 10.1002/cns.70716 (PMC12723193; doi:10.1002/cns.70716)
Supplement: Supplementary file 1 — Table S1: cns70716‐sup‐0001‐AppendixS1.docx. [file CNS-31-e70716-s001.docx]

**Table S1. The origin of the crude drugs in NaoFuCong (NFC) decoction**

| **Herb** | **Latin name** | **Species** | **Amount (g)** | **Batch numbers** | **Major constituents** |
| --- | --- | --- | --- | --- | --- |
| Ginseng (Ren-Shen) | Panacis ginseng radix | Panax ginseng C.A.Mey. | 6 | D6092221 | Ginsenosides; Ginsenic acid |
| Danshen (Dan-Shen) | Salviae miltiorrhizae radix | Salvia miltiorrhiza Bunge | 30 | D0246371 | Tanshinones; Salvianolic acids |
| Prepared Polygonum (Zhi-He-Shou-Wu) | Polygoni multiflori radix Praeparata | Polygonum multiflorum Thunb. | 30 | D526A531 | Emodin; Polygonum multiflorum saponins |
| Processed Leech (Tang-Shui-Zhi) | Hirudo medicinalis | Hirudo nipponica Whitman, Whitmania pigra Whitman, etc. | 12 | C6032132 | Hirudin; Proteases |
| Poria (Fu-Ling) | Poria | Poria cocos (Schw.) Wolf | 30 | C026G341 | Pachymic acid; Poroid |
| Coptis (Huang-Lian) | Coptidis rhizoma | Coptis chinensis Franch., Coptis deltoidea C.Y.Cheng et Hsiao, etc. | 20 | D517B511 | Berberine; Coptisine |
| Acorus (Shi-Chang-Pu) | Acori tatarinowii rhizoma | Acorus tatarinowii Schott | 20 | D9091431 | α - Asarone; β - Asarone |

**Table S2. The antibody used in IF**

| **Antibody type** | **Antibody name** | **Dilution ratio** | **supplier** | **Art.No.** |
| --- | --- | --- | --- | --- |
| primary antibody | GFAP | 1:1000 | Servicebio | GB12096 |
| primary antibody | Iba1 | 1:200 | Abcam | ab5076-1001 |
| primary antibody | NeuN | 1:5000 | Gene Tex | GTX00837 |
| secondary antibody | DAPI | 1:400 | Solarbio | C0060 |
| secondary antibody | 647 | 1:400 | ThermoFisher | A-21449 |
| secondary antibody | 568 | 1:10000 | Abcam | ab175474 |
| secondary antibody | 488 | 1:400 | Yesen | 34106ES60 |

**Table S3. Primer sequences for qPCR**

| **Gene** | **Forward primer (5′-3′)** | **Reverse primer (5′-3′)** |
| --- | --- | --- |
| **BDNF** | AAG GCA CTG GAA CTC GCA ATG | TTA TGA ACC GCC AGC CAA TTC TC |
| **NGF** | CAT CCA CCC ACC CAG TCT TCC | TCC GTG GCT GTG GTC TTA TCT C |
| **SYN** | GCT CAT CGT GGA ACT TGT GGT C | ATG GAG TCT GGC TGT GGG AAC |
| **PSD-95** | CAC AGA GTG CTT CTC AGC CA | TAG GGG CCT GAG AGG TCT TC |
| **β-actin** | CTA TCG GCA ATG AGC GGT TCC | GCA CTG TGT TGG CAT AGA GGT C |
| **qPCR reaction procedure is as follows:**  (1) Holding stage: 95°C for 20 seconds;  (2) PCR stage: 95°C 1 second, 60°C 20 seconds, a total of 40 cycles;  (3) Melting curve stage: 95 C 1 second, 60°C 20 seconds, 95 C 1 second. | | |

**Table S4. The antibody used in WB**

| **Antibody type** | **Antibody name** | **Dilution ratio** | **supplier** | **Art.No.** |
| --- | --- | --- | --- | --- |
| primary antibody | BDNF | 1:1000 | Abcam | ab108319 |
| primary antibody | NGF | 1:2000 | HUABIO | ET1606-29 |
| primary antibody | SYN | 1:1000 | Abcam | ab254349 |
| primary antibody | PSD-95 | 1:2000 | Abcam | ab238135 |
| primary antibody | APP | 1:6000 | Abcam | ab126732 |
| primary antibody | p-Tau | 1:1000 | Abcam | ab92676 |
| primary antibody | PKA | 1:1000 | Selleck | F0588 |
| primary antibody | P-PKA | 1:1000 | Selleck | F1206 |
| primary antibody | CREB | 1:1000 | Selleck | F0133 |
| primary antibody | P-CREB | 1:5000 | Selleck | F2525 |
| secondary antibody | IgG Antibody | 1：50000 | HUABIO | HA1001 |

**Untargeted metabolomics analysis**

For liquid chromatography (LC) separation, samples were analyzed using a ACQUITY UPLC® HSS T3 column （2.1×100 mm, 1.8μm）(Waters, Milford, MA, USA). The flow rate was 0.3 mL/min and the mobile phase contained: A: 0.1% FA in water and B: 100% acetonitrile (ACN). The gradient was 0% buffer B for 2 min and was linearly increase to 48% in 4 min, and then up to 100% in4 min and maintained for 2 min, and then decreased to 0% buffer B in 0.1 min, with 3 min re-equilibration period employed.

The HESI source conditions were set as follows: Spray Voltage：3.8kv (positive) and 3.2kv (negative)；Capillary Temperature：320 ℃; Sheath Gas (nitrogen) flow: 30 arb (arbitrary units); Aux Gas flow: 5 arb; Probe Heater Temp: 350 ℃; S-Lens RF Level：50. The instrument was set to acquire over the m/z range 70-1050 Da for full MS. The full MS scans were acquired at a resolution of 70,000 at m/z 200, and 17,500 at m/z 200 for MS/MS scan. The maximum injection time was set to for 100 ms for MS and 50 ms for MS/MS. The isolation window for MS2 was set to 2 m/z and the normalized collision energy (stepped) was set as 20, 30 and 40 for fragmentation.
